# Benchmarking progression-free survival ratio as primary endpoint in precision oncology clinical trials

**DOI:** 10.1038/s41698-025-01231-x

**Published:** 2025-12-15

**Authors:** Federico Nichetti, Jennifer Hüllein, Pauline du Rusquec, Erin Pleasance, Li Chen, Andreas Mock, Peter Horak, Paolo Ambrosini, Simone Rota, Monica Niger, Luca Agnelli, Gabriele Tinè, Dominic Edelmann, Christophe Le Tourneau, Janessa Laskin, Giancarlo Pruneri, Chi Wang, Daniel Hübschmann, Filippo de Braud, Stefan Fröhling, Luigi Mariani

**Affiliations:** 1 Oncology Unit 1, Veneto Institute of Oncology IOV - IRCCS, Padua, Italy; 2 Computational Oncology, Molecular Precision Oncology Program, National Center for Tumor Diseases (NCT) and German Cancer Research Center (DKFZ), Heidelberg, Germany; 3 Department of Drug Development and Innovation (D3i), Institut Curie, Paris, France; 4 INSERM U900 Research unit, Institut Curie, Saint-Cloud, France; 5 Canada’s Michael Smith Genome Sciences Centre, BC Cancer, Vancouver, BC Canada; 6 Division of Cancer Biostatistics, Department of Internal Medicine, University of Kentucky, Lexington, KY USA; 7 Markey Cancer Center, University of Kentucky, Lexington, KY USA; 8 Institute of Pathology, University of Munich (LMU), Munich, Germany; 9 German Cancer Consortium (DKTK), Heidelberg, Germany; 10 Division of Translational Medical Oncology, National Center for Tumor Diseases (NCT) Heidelberg, German Cancer Research Center (DKFZ), Heidelberg, Germany; 11 Medical Oncology Department, Fondazione IRCCS Istituto Nazionale dei Tumori di Milano, Milan, Italy; 12 Department of Advanced Diagnostics, Fondazione IRCCS Istituto Nazionale dei Tumori di Milano, Milan, Italy; 13 Biostatistics for Clinical Research Unit, Epidemiology and Data Science Unit, Fondazione IRCCS Istituto Nazionale dei Tumori di Milano, Milano, Italy; 14 Division of Biostatistics, German Cancer Research Center (DKFZ), Heidelberg, Germany; 15 NCT Trial Center, National Center for Tumor Diseases, Heidelberg, Germany; 16 Paris-Saclay University, Paris, France; 17 Department of Medical Oncology, BC Cancer, Vancouver, BC Canada; 18 Department of Oncology and Hemato-Oncology, University of Milan, Milan, Italy; 19 Pattern Recognition and Digital Medicine Group, Heidelberg Institute for Stem cell Technology and Experimental Medicine (HI-STEM), Heidelberg, Germany

**Keywords:** Computational biology and bioinformatics, Oncology, Mathematics and computing

## Abstract

Progression Free Survival Ratio (PFSratio), as defined as the ratio between PFS on investigational treatment (PFS2) and PFS on the last prior therapy (PFS1), is a popular endpoint in precision oncology (PO) studies. In this work, five methodologies for PFSratio-based trial analysis (count-based, Kaplan Meier, Kernel-based Kaplan Meier, parametric and midrank) and two for trial design (GBVE and Weibull) are benchmarked. The Kernel-based Kaplan Meier analysis is most recommended, as it handles informative censoring and does not require PFS1/PFS2 distribution assumptions. Sample size and power calculation methods perform best when applied to settings with expected high PFS1/PFS2 correlation and median ratio. Analysis of five clinical trials (MOSCATO 01, WINTHER, MASTER, SHIVA and POG570) from >800 patients revealed an overall weak PFS1/PFS2 correlation (Kendall’s *τ* range 0.17-0.35), and an asymptotically unbiased median *S*_PFSratio_(*δ*=1.3) = 33% by means of the Kernel-based analysis, while other methods considerably deviated in studies with censoring rate>10%. This methodology is implemented in the *PROPHETS* R package and Shiny app.

## Introduction

Over the last decades, the widespread use of next generation sequencing (NGS) technologies has led to the advent of precision oncology (PO), defined as the use of tumor molecular profiling to tailor anticancer treatment. Molecularly informed therapies have indeed often demonstrated better efficacy and tolerability than standard chemotherapy in clinical trials and in clinical practice^1^.

Typically, targeted therapies are initially tested in advanced treatment line settings when standard options are exhausted or have a low probability of success. Specifically, phase II trials represent the cornerstone to look for clinically significant activity of new compounds or combinations. However, most druggable molecular alterations are rare, with varying prevalence and actionability according to each tumor type. Moreover, in advanced treatment lines, the use of placebo- or low effect standard of care-based control arms is ethically questionable. These features prevent from rapidly conducting numerically large phase II trials, especially randomized ones. Furthermore, in the lack of appropriate historical controls, single-arm phase II studies can be burdened by great interpatient and between-trial variability, so that the results are often difficult to generalize. These considerations are leading to a paradigm shift in PO phase II trials, with novel adaptive design and new endpoints being implemented in recent years^2^.

In this light, the Progression Free Survival Ratio (PFSratio) has been initially proposed by Von Hoff^3^ as a novel endpoint for trials investigating new anticancer agents in patients who have failed at least one previous regimen. The PFSratio, also known as time to progression ratio (TTPr) or growth modulation index (GMI) is defined as the ratio between PFS on the therapy under investigation (PFS2) and the PFS on the last prior therapy (PFS1). This endpoint has some advantages compared to those usually adopted in phase II trials, like objective response rate or PFS, since it allows a) reduction in heterogeneity, as each patient serves as his/her own control, b) design of single-arm trials with limited sample size, and c) provision of a clinically relevant estimate of benefit of a new treatment. In recent years, a detailed exploration of PFSratio statistical properties has been performed, to correctly adopt it for the design and subsequent analysis of a trial^4–7^, but the methods remained scattered and difficult to interpret for clinicians. Moreover, several PO trials explored PFSratio as a measure of benefit, but different methods have been adopted, making the results poorly comparable.

In this work, we summarize current knowledge on PFSratio use in PO clinical trials, describing its clinical and statistical characteristics. Furthermore, we collect and implement different methods for the design and analysis of trials that use PFSratio as their primary endpoint. Finally, we explore PFSratio on outcome data of major PO clinical trials, providing a uniform analysis for proper interpretation of this methodology and corresponding results.

## Results

**Fig. 1:**
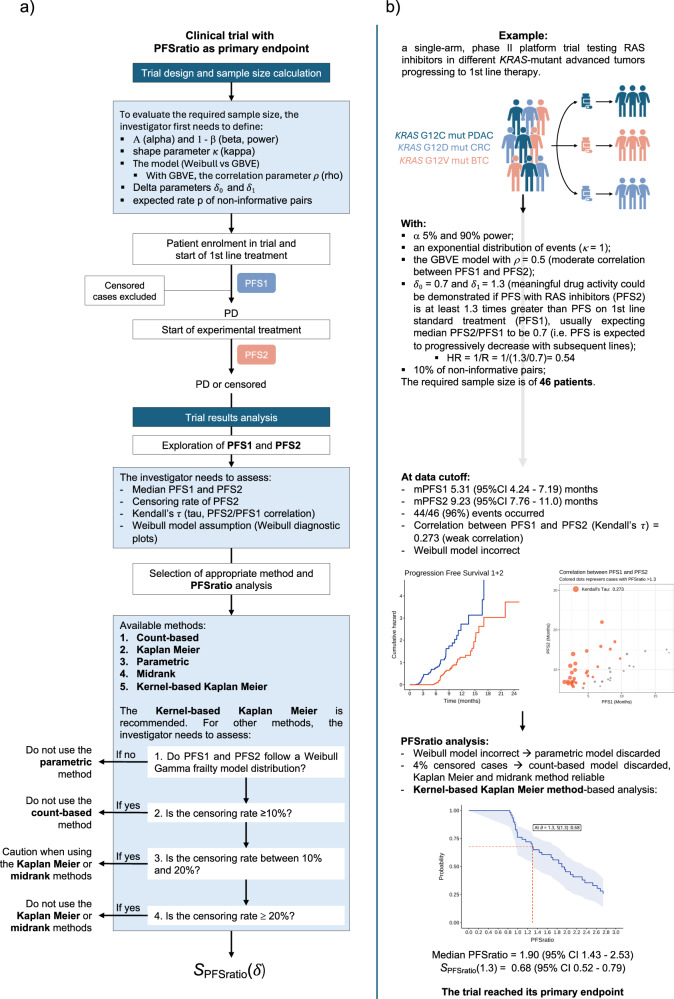
PFSratio workflow. **a**, **b** Proposed workflow (a) and worked example (b) to properly design, conduct and analyze a clinical trial with PFSratio as its primary endpoint. A single-arm, phase II platform trial testing novel RAS inhibitors in different *KRAS*-mutant advanced tumors progressing to 1st line therapy is hypothesized, and results are generated through a simulation with randomly generated data. The first step is trial design and sample size calculation. Then, the optimal setting is a to start enrolling patients at 1st line treatment initiation, following the same tumor assessment schedule during PFS1 and PFS2. Once the study has adequate follow up, exploration of PFS1 and PFS2 is recommended, followed by PFSratio analysis selecting the proper method to avoid biased results. Abbreviations: BTC biliary tract cancer, CI confidence interval, CRC colorectal cancer, GBVE Gumbel’s type B bivariate extreme-value, kappa shape of the hazard function, KM Kaplan Meier, PFS progression free survival, PFSratio progression free survival ratio, PD progressive disease, PDAC pancreatic ductal adenocarcinoma, rho pearson correlation index between PFS1 and PFS2, R median ratio between PFS1 and 2, detailed with corresponding *δ*_1_ and *δ*_0_.

A worked example on how to properly design, conduct and analyze a clinical trial with PFSratio as its primary endpoint is provided in Fig. 1.

In clinical research, trial design with sample size calculation is the natural first step, while analysis of study results occurs only after its completion. In this work however, to guide the reader to the understanding of PFSratio methodology, we first describe the methods for PFSratio analysis and only later those for PFSratio-based trial design and sample size calculation.

The scenario considered here is that of a phase II trial that evaluates clinical benefit by comparing, within each treated patient, the failure time observed for a new agent under investigation with the failure time observed in the last prior line of treatment. The failure time is typically progression free survival (PFS), and the pair of values available for each patient is denoted as PFS1 and PFS2 for the two chronologically ordered treatment lines. Typically patients cannot be censored on PFS1, as only the patients who have progressed on the prior line of treatment will move to the subsequent line of treatment, but are censored on PFS2 whenever follow-up stops before progression for whatever reason, like the occurrence of censoring events (e.g. toxicity) or when the end of study has been reached. Note that, for PFSratio as an endpoint to be unbiased, the gold standard setting for its use is a clinical trial in which both PFS1 and PFS2 are assessed prospectively and in a consistent manner -that is, using the same criteria (radiological and/or clinical) and at the same evaluation frequency^8^.

### Exploration of paired failure times

Before PFSratio analysis, individual assessment of PFS1 and PFS2 is essential. In detail, it is of primary importance to assess the pattern of each PFS, the censoring rate of PFS2, and possibly the correlation between the two-paired PFS outcomes.

Descriptive analyses of PFS1 and PFS2 separately can be performed with conventional tools, like the Kaplan Meier method for plotting survival curves, while censoring rate is easily calculated as the proportion of patients with censored PFS2 out of the total number. Estimating the correlation between PFS1 and PFS2 is a less easy task, as it involves the use of frailty models. In fact, positive correlation is likely to exist, as PFS1 and PFS2 may be affected not only by treatment efficacy, but also by other factors which are characteristics of each patient^6^. For example, patients with indolent tumors tend to stay longer under treatment, independently of therapy line, so that longer PFS1 corresponds to longer PFS2 or vice versa. However, this assumption does not always hold true^9^, especially in PO trials where a targeted agent may provide a significantly different benefit than the previous standard treatment, and must be therefore verified.

Regarding frailty models, they are extensions of conventional survival models that include additional “frailty” parameters that handle the correlation. One such model that is widely considered in this context is the Weibull Gamma frailty model, so named because the frailty term is distributed according to a gamma distribution, while, conditional on the frailty term, failure times PFS1 and PFS2 are assumed to follow a Weibull distribution. Interestingly, by denoting the frailty term with β*θ*, this model allows estimating the intrapatient dependence (i.e. the correlation between PFS1 and PFS2) via Kendall’s *τ*, with *τ*= *θ*/(*θ*+2). The calculation of this correlation index is crucial as it affects the performance of PFSratio in the analysis phase and sample size calculation in study design, as shown later.

The Weibull part of the model contains instead two parameters, one modeling the divergence between PFS1 and PFS2, the other one (*K*) the shape of the hazard function, that is the rate at which the events occur over time. A value of this parameter >1 ( < 1) denotes a rate that is increasing (decreasing) over time, while a value equal to one implies an exponential distribution of failure times, characterized by a failure rate that is constant. These properties make the Weibull Gamma frailty model sufficiently flexible to fit most of the situations that we encounter in practical situations. A way to check the Weibull assumption is to plot the logarithm of the cumulative hazard, i.e. log[- log(S(t)] where log stands for the natural logarithm and S(t) is the Kaplan Meier survival estimate for PFS, against log(survival time). The Weibull assumption is correct if this plot gives two parallel and approximately straight lines for PFS1 and PFS2.

This exploration, along with considerations regarding censoring rates (discussed below), is necessary to apply the correct method for PFSratio analysis. In particular, among the methods described below, the parametric method can be properly applied only if PFS1 and PFS2 follow a Weibull distribution^10^.

### Analysis of PFSratio

As the name implies, PFSratio is calculated as PFS2/PFS1. In this way, a pair of values is transformed into a single but possibly censored, non-negative quantity, and a clinical study will provide N independent ratios computed from the pairs (PFS1, PFS2).

To judge the goodness of this ratio, a reference *δ* reflecting minimal improvement in PFS with the investigated treatment needs to be set by the investigator. The *δ* parameter represents the PFSratio cutoff that defines the investigational treatment as “effective” / the individual patient as “responder” if his/her PFSratio is no less than, or “ineffective” / “non responder” otherwise. A quite straightforward choice is *δ*=1, because this value is exceeded whenever PFS2 is greater than PFS1, which is clearly a sign of therapeutic improvement. As more fully described below, however, other criteria for choosing *δ* may be followed. Whatever the choice of *δ*, a study that is based on PFSratio as an efficacy endpoint is aimed at estimating the probability that this ratio is equal to or greater than *δ*, i.e. *P*(PFSratio ≥ *δ*), or *S*_PFSratio_(*δ*) more compactly. *S*_PFSratio_(*δ*) (and its confidence interval, CI) thus represents the probability of having a ratio > *δ*, which can be interpreted as the fraction of patients from a given cohort with a clinically relevant improvement of PFS2 relative to PFS1^5,6^.

To further determine the efficacy of the investigational treatment in a patient population, a targeted minimal proportion of patients for whom the treatment is effective, i.e., patients with PFSratio ≥ *δ*, needs to be set by the investigator. We refer to p_0_ as the targeted minimal proportion. A study is supportive of treatment efficacy whenever the probability of PFSratio ≥ *δ*, i.e., *S*_PFSratio_(*δ*), together with its lower CI, is shown to be greater than p_0_. The choice of p_0_ varied across different studies. In MOSCATO 01^11^, p_0_ was set to be 15%. In WINTHER^12^, p_0_ was set to be 40% or 50%.

Several methods have been proposed to estimate *S*_PFSratio_(*δ*), namely the count-based, Kaplan Meier, parametric, midrank (non-parametric) and a novel kernel-based Kaplan Meier, individually discussed below.

Note that, when selecting methods for PFSratio analysis, PFS2 censoring rates must be taken into account. We recommend using PFSratio as an endpoint when the censoring rate for PFS2 is below 50%, as estimate accuracy may otherwise decline -especially with higher *δ* values and/or smaller median PFSratios. The kernel-based Kaplan-Meier method must be preferred when the censoring rate is ≥20%, as detailed below.

Most importantly, interpretation of PFSratio must also consider the clinical context, including the chosen *δ* value and the cohort follow-up. Indeed, even with high censoring, censored cases with PFSratio > *δ* remain informative.

### Count based method

The simplest estimator for *S*_PFSratio_(*δ*) is based on the count of PFSratio outcomes equal to or greater than *δ*. For the proportion so obtained, a confidence interval and a statistical test can be constructed. A limitation of this method is that all those pairs for which PFSratio < *δ* and PFS2 is censored are not informative. The reason is that actual PFS2, if known, could take values that make the ratio greater or less than *δ*, thus generating uncertainty on how to handle these pairs. For this reason, they must be excluded from the calculation (unlike what happens with all other methods), with a loss of information as a consequence.

### Kaplan Meier method

The PFSratio can be regarded as the value of PFS2 measured in terms of PFS1 units: if PFS2 is censored, the PFSratio is censored too. For this reason, PFSratio can be treated as a time-to-event variable as the probability of surviving (or not progressing) at a given timepoint *δ* and survival analysis methods can be applied for its evaluation. Among them, the Kaplan Meier method allows the investigator to easily obtain an estimate of *S*_PFSratio_(*δ*) with the corresponding CI. Furthermore, the Kaplan Meier survival curve describing the distribution of PFSratios in the overall study cohort can be plotted, and estimates of the median PFSratio and its corresponding CI can be straightforwardly obtained.

### Parametric method

It has been shown that if PFS1 and PFS2 follow the Weibull Gamma frailty model, their ratio follows a log-logistic distribution^6,13^. Using this distribution, the investigator can obtain maximum likelihood estimates of the distribution parameters from the possibly censored PFSratio data and derive *S*_PFSratio_(*δ*) by plugging these estimates into a simple formula.

### Midrank method

This method, as is typical of non-parametric tools, aims to compare the distributions of two independent variables without making strong statistical assumptions on their shape. In our specific context, the approach consists in using the ranks of each pair (PFS1, PFS2) to estimate *S*_PFSratio_(*δ*). Due to censoring, the ranks of some observations are unknown but can be estimated by midranks according to the procedure proposed by Hudgens and Satten^5,6,14^. However, this midrank method requires the assumption that the censoring distributions for PFS1 and PFS2 are the same, which is violated because PFS1 is uncensored while PFS2 is censored^14,15^.

### Kernel-based Kaplan Meier method

Recently Chen et al.^15^ recognized that, unlike the censoring of PFS2, which can usually be assumed to be uninformative, the censoring of PFSratio is always informative regardless of whether PFS2 is uninformatively censored or not. In detail, let C2 denote the time to the censoring events, such as toxicity or ending of study, corresponding to PFS2. As a result, the censoring variable corresponding to PFSratio, PFS2/PFS1, is C2/PFS1. Since both PFSratio and its censoring variable involve PFS1, they are dependent, which violates the uninformative (or independent) censoring assumption required by common survival analysis methods like those previously described. Consequently, novel parametric and non-parametric kernel-based Kaplan Meier methods were proposed for estimating the *S*_PFSratio_(*δ*) function and the corresponding confidence interval while adequately accounting for informative censoring. Unlike the Kaplan-Meier estimator, which directly estimate *S*_PFSratio_(*δ*), i.e. the survival function of PFSratio, the kernel-based Kaplan-Meier estimator utilizes a two-stage procedure. In the first stage, it utilizes a kernel conditional Kaplan-Meier method to estimate the conditional survival function of the PFSratio given PFS1. In the second stage, it averages these conditional survival functions across all observed PFS1 values. Since the first stage requires only the assumption that the PFSratio is conditionally independently censored given PFS1, and the second stage imposes no additional assumptions, the consistency of the estimator relies solely on this conditional independent censoring assumption. Based on standard statistical theory, this assumption is satisfied if PFS2 is independently censored, a common and often reasonable condition in time-to-event analyses. Regarding the kernel function used in the estimator, Chen et al.^9^ employed a modified Silverman Kernel, which is flatter than the Gaussian kernel and reduces the risk of near-zero values in the denominator, to ensure the numerical stability.

The authors proved theoretically and by means of extensive simulations that this new estimator is asymptotically unbiased. In contrast, previous methods were shown to be biased not only in simulations considering a censoring rate of 20% or 30%, but also in cases when the censoring rate was between 10% and 20%, especially for sample size ≥ 100 (data not reported).

Since the novel kernel-based non-parametric method holds the favorable features previously illustrated for the simple Kaplan Meier, while also accounting for dependent censoring, it will be used in the following sections as the reference method for trial analysis.

### Modified PFSratio

Concerning the duration of PFS1 and PFS2, a careful evaluation is recommended to avoid under- or over-estimation of PFSratio in cases of very short or long PFS1, respectively. To address this issue, Mock et al. proposed a modified PFSratio (mPFSratio), where PFS1 shorter than 2 months converts to 2 months and PFS2 longer than 6 months converts to 24 months before ratio calculation^16^. The underlying logic is that patients rapidly progressing with previous treatment, e.g. PFS1 of one month, might have an overly optimistic PFSratio even in case of short PFS2. Conversely, a sufficiently long PFS2, e.g. greater than six months, should in itself indicate sufficient activity of the experimental treatment. While the conversion of extremely short PFS1 to 2 months is reasonable in most cases, PFS2 conversion from >6 to 24 months is debatable. In particular, 6 months can be taken as a clinically meaningful cutoff to consider a PFS2 treatment as effective, but it may change according to the clinical context and the chosen *δ*. To address this issue, we propose a simple alternative method where PFS1 is equally transformed to 2 months in cases with extremely short ( < 2 months) PFS1, while PFS2 is transformed as follows:

- first, the clinical investigator decides what is the minimum PFS2 that can be considered satisfying irrespective of PFS1 (e.g., 6 months);
- then, once *δ* is defined, in cases where PFS2 is satisfying but PFSratio is < *δ*, PFS2 is transformed as PFS1 * *δ* + 0.25, with 0.25 representing a minimum advantage (~1 week, with survival estimates calculated in months) to PFS2 to avoid random fluctuations.

The adjustments to PFS1 and PFS2 reduce sensitivity to very small denominators and dampen outlier influence without imposing parametric structure. This leads to more consistent analysis results across the different estimators evaluated as compared to the results based on original PFSratio (see Supplementary Table 1).

Clearly, the use of the modified PFSratio must be even more strongly guided by the clinical context under consideration, in order to define precisely what duration of PFS2 should be regarded as clinically meaningful, and to account for situations in which PFS1 may appear extremely short (e.g., clinical trials with CT scans scheduled every 6 weeks, which can result in very brief PFS1 estimates). Ultimately, since this is a descriptive, clinically derived endpoint rather than one built on a formal statistical model, further statistical validation will be required in this context.

### Sample size calculation

Sample size calculation is a mandatory early step when designing a clinical trial, and PFSratio-based studies are no exception.

Once excluding simulation, which is generally possible but not easily doable, a handy solution is based on the use of analytical formulae, as proposed by Wu et al.^7^. In practice, having defined the desired levels of significance (alpha) and power (1-beta), the calculation is based on the so-called Generalized Treatment Effect (GTE), that is the probability *S*_PFSratio_(*δ*) expected under the hypothesis of treatment efficacy. This probability can be specified directly, according to what the authors define as “Study design using generalized effect size”. More accurately, a GTE value can be derived on the basis of the assumptions regarding the distribution of PFS1 and PFS2.

One possibility is to assume the Weibull-Gamma frailty model previously described. With this model, specific values must be hypothesized for the shape parameter *K* and the ratio R, i.e., the median ratio of PFS2 versus PFS1. With *K* = 1, i.e., in case of exponential distribution, R corresponds to the reciprocal of the hazard ratio of PFS2 vs PFS1, HR = 1/R.

As an alternative, a bivariate exponential model, namely the Gumbel’s type B bivariate extreme-value (GBVE) model, may be assumed, in which both PFS1 and PFS2 follow an exponential distribution. In this case, the inputs required are the Pearson correlation coefficient *ρ* between PFS1 and 2 and the ratio R. Mick and colleagues^4^ first showed that the *ρ* parameter is crucial: the stronger the correlation, the greater the power of the study and the smaller the sample size. Of note, a negative correlation (*ρ* < 0) between PFS1 and 2 (i.e., settings in which a longer PFS2 corresponds to shorter PFS1 or vice versa) is clinically unlikely. Finally, regardless of the model assumed for sample size calculation, it is necessary to specify two values for *δ*: one is the value assumed under the null hypothesis, *δ*_0_, which has the previously explained meaning of the cutoff that defines the starting point of effectiveness, the other is the value assumed under the alternative hypothesis, *δ*_1_, which represents the level of effectiveness at which the desired study power is achieved.

For example, PO trials aiming at demonstrating a meaningful benefit of targeted treatments after a prior standard therapy are usually designed with *δ*_0_ = 1 and slightly greater *δ*_1_ (e.g., 1.3 or 1.5), while in settings where PFS is expected to progressively decrease with subsequent lines of treatment, a PFS2 equal to PFS1 can be considered satisfactory, and therefore *δ*_0_ can be set slightly below one (e.g., 0.7) and *δ*_1_ = 1.

Once the GTE has been specified or derived by either of the above two methods, the required number n of paired events can be calculated. To obtain the sample size N, i.e., the total number of pairs, adjustment of n by the expected rate p of non-informative pairs, that being the rate of cases with PFSratio < *δ* and censored PFS2, based on the formula: N = n / (1 – p), is required. If the trial is designed with a long enough follow-up time, the p-rate is small and the correction is minimal, usually around a few percentage points.

**Fig. 2:**
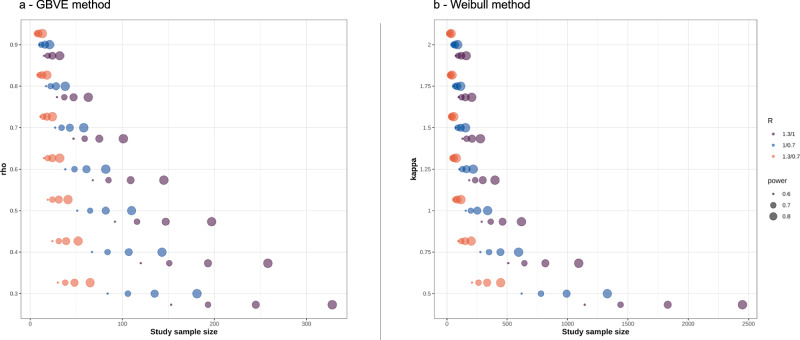
PFSratio-based clinical trial design. **a**, **b** Graphic representation of the impact of correlation (rho), power, HR on sample size in PFSratio-based clinical trial design. Abbreviations: GBVE Gumbel’s type B bivariate extreme-value, kappa shape of the hazard function, rho pearson correlation index between PFS1 and PFS2, R median ratio between PFS1 and 2, detailed with corresponding *δ*_1_ and *δ*_0_.

Figure 2a shows the impact of different *ρ*, power and R on the required N adopting the GBVE model: greater *ρ* values as well as greater R result in smaller patient numbers, while greater power requires larger numbers. From a clinical point of view, this exploration also shows that in settings with weak correlation (e.g., < 0.3), possibly combined with R close to one (small effect size), the required sample size becomes very large and makes it difficult to conduct the trial in a reasonable amount of time. Conversely, in contexts of expected clear superiority of PFS2 over PFS1 and with moderate/strong correlation between the two, a clinical study can be adequately conducted with only 20–30 patients. Regarding the Weibull model, Wu et al. previously showed that increasing *K* values result in smaller sample size^7^, while *ρ* is not directly considered in the calculation. For simulations presented in Fig. 2b, we show that higher *K*, power and R similarly result in smaller study sample size, while extremely high numbers (and thus not realistic for a PO trial) are required if *K* < 1.

An easy-to-use interface for the clinician to calculate the study sample size or power is available at https://federico-nichetti.shinyapps.io/prophetsshiny/. In addition, since many works explored PFSratio as a secondary analysis, investigators may be interested in testing the power of the analysis if N is known. To this aim, we implemented the reverse calculation, both with a GBVE and a Weibull model. Supplementary Fig. 1a, b show that, given a fixed sample size, studies with greater R (i.e. *δ*_0_ < 1 and *δ*_1_ > 1) keep a high power which is less affected by *ρ* and *K* in the GBVE and a Weibull models, respectively.

### PFSratio-based PO trials survey

Data from five PO trials were collected and compared in the PFSratio-based analysis, namely MOSCATO 01^11^, WINTHER^12^, MASTER^17^, SHIVA^18^, and POG570^19^. All of these studies (except for POG570) previously adopted PFSratio as an endpoint, primary or secondary, and all shared the common feature of genome- and transcriptome-based treatment selection in advanced cancer patients. A summary of these studies is provided in Table 1.

**Table 1:** Summary of PO clinical trials tested in the PFSratio-based analysis

Study	year(s)	Site	Tumor types treated	Type of molecular screening	Primary endpoint	PFSratio analysis	n° of patients evaluable for PFSratio
**WINTHER (NCT01856296)**	2013–2015	Multicentric, international	Advanced cancers that progressed on standard Tx	Targeted genomic (236 cancer-related genes panel) + transcriptomic (microarrays)	PFSratio	Proportion of patients with PFSratio >1.5	107
**MASTER (NCT03127215)**	2012–ongoing	Multicentric, national	Advanced rare cancers or cancers in young patients ( < 51 years) that progressed on standard Tx	WGS/WES + transcriptomic (RNAseq)	Feasibility and clinical relevance of comprehensive molecular analysis in rare cancers	Proportion of patients with PFSratio >1.3	255
**MOSCATO 01 (NCT01566019)**	2011–2016	Monocentric	Advanced cancers that progressed on standard Tx	Targeted genomic (40-75 cancer-related genes panel) or WES + transcriptomic (RNAseq)	PFSratio	Proportion of patients with PFSratio >1.3	194
**POG570 (NCT02155621)**	2012– ongoing	Monocentric	Advanced cancers that progressed on standard Tx	WGS + transcriptomic (RNAseq)	Feasibility and clinical relevance of comprehensive molecular analysis in advanced cancers	Not done	190
**SHIVA (NCT01771458)**	2012–2014	Multicentric, national	Advanced cancers that progressed on standard Tx	Targeted genomic (45 cancer-related genes panel) + IHC for ER, PR and AR	PFS	Proportion of patients with PFSratio >1.3	95

*AR* androgen receptor, *ER* estrogen receptor, *IHC* immunohistochemistry, *PFSratio* progression free survival ratio, *PR* progesterone receptor, *Tx* treatment, *WES* whole exome sequencing, *WGS* whole genome sequencing.

**MOSCATO 01** (Molecular Screening for Cancer Treatment Optimization) was the first, monocentric, PO trial using targeted and whole exome (WES) plus transcriptome profiling to tailor treatment and adopting PFSratio as its primary endpoint^11^; **WINTHER** was an international, non-comparative trial in which genomic (via a 236 cancer-related genes panel, arm A) or transcriptomic (arm B) data were used to propose targeted therapies, which failed to meet its primary endpoint of PFSratio >1.5 in 50% and 40% of arm A and B patients, respectively^12^. **MASTER** (Molecularly Aided Stratification for Tumor Eradication Research) is a multicentric registry trial and analytical platform established in 2012 by the German Cancer Consortium (DKTK) for prospective, multi-omics-guided stratification of patients with advanced cancers diagnosed at a young age ( < 51 years) or with rare cancers, and comprises whole-genome/whole-exome sequencing (WGS/WES) and transcriptome profiling^20^. While the study is ongoing, the data presented here refer to the first published cohort treated up to November 2018 with available PFSratio data^17^. **SHIVA** was a multicentric, randomized phase II trial comparing treatment of physician choice (TPC) to molecularly-targeted agents (MTA, i.e., drugs that were approved for clinical use in France, but outside their indication) in patients with refractory cancers, allowing for crossover in both study arms^21^; here we applied PFSratio methods in the 95 patients who crossed-over, as previously reported^18^. Data analysis was performed separately on the TPC - > MTA and in the MTA - > TPC cohorts. **Personalized OncoGenomics (POG)** is a clinical trial conducted at BC Cancer (Canada) investigating the feasibility and utility of WGS and transcriptome analysis for informing targeted treatment of patients with advanced tumors. For this work, we analyzed for the first time the PFSratio of patients with available PFS1 and PFS2 data within the first 570 treated cases (POG570)^19^, with only the first targeted treatment considered in patients that received more than one molecularly guided therapy.

For this work, MOSCATO 01 PFS data were reconstructed from original paper plots using the *digitize* R package and results were double checked with data reconstructed using the *Inkscape* software; WINTHER data were retrieved, after formal request, from supplementary data of the original work^12^ using the *pdftables* R package; MASTER data were shared after formal project approval at the DKTK MASTER Scientific Board meeting. SHIVA and POG570 data were shared after formal request to the corresponding authors of the original paper (SHIVA: P.d.R. and C.L.T., POG570: E.P. and J.L.)

Among the explored studies, a total of 841 patients were included, with as many pairs of PFS1 and 2. Details concerning each study are reported in Table 1. All studies included pretreated patients with advanced cancers, with varying sequencing coverage for molecular characterization, ranging from small targeted genomic panels to WGS and transcriptome sequencing. As shown, with the exception of POG570, these studies analyzed PFSratio simply as the proportion of cases with PFSratio > 1.3 or > 1.5.

We re-analyzed these trials according to the above-described methods. A summary of the analysis is provided in Table 2.

**Table 2:** Summary of PFS1, PFS2 and PFSratio analysis of precision oncology trials

Study	Median PFS1 (months, 95% CI)	Median PFS2 (months, 95% CI)	Median PFSratio	Weibull distribution	Kendall’s Tau	Proportion of informative cases	_*S*PFSratio_(*δ*=1.3)
**WINTHER**	4.1 (3.2–5.2)	2.0 (1.8–3.1)	0.74 (0.57–0.83)	no	0.314	106/107 (99%)	0.25 (0.17–0.34)
**MASTER**	3.2 (3.0–3.7)	3.5 (3.1–4.1)	1.02 (0.86–1.21)	no	0.220	233/255 (91%)	0.41 (0.34–0.47)
**MOSCATO 01**	3.0 (2.7–3.6)	2.4 (1.9–2.8)	0.75 (0.58–0.92)	no	0.174	193/194 (99%)	0.33 (0.27–0.40)
**POG570**	3.5 (2.8–4.1)	3.4 (2.5–4.3)	0.81 (0.60–1.01)	no	0.228	152/190 (80%)	0.33 (0.26–0.41)
**SHIVA (TPC -** > **MTA)**	2.0 (1.9–.4)	2.3 (2.1–4.1)	1.03 (0.87–1.55)	no	0.295	61/70 (87%)	0.42 (0.29–0.54)
**SHIVA (MTA -** > **TPC)**	2.8 (2.1–4.0)	2.3 (1.8–3.2)	0.67 (0.52–1.04)	no	0.354	24/25 (96%)	0.21 (0.08–0.39)

Median PFSratio and *S*_PFSratio_(*δ*=1.3) were calculated using the kernel-based Kaplan Meier method.

*CI* confidence interval, *MTA* molecularly targeted agent, *PFS* progression free survival, *PFSratio* progression free survival ratio, *TPC* treatment of physician’s choice, S_PFSratio_ the probability that PFSratio is equal to or greater than *δ.*

First, individual assessment of paired PFS1 and PFS2 was performed. Median PFS1 ranged from 2.0 to 4.1 months, while PFS2 from 2.0 to 3.5 months, with a censoring rate on PFS2 ranging from 1% to 20% (see Supplementary Fig. 2a–f).

Of note, Weibull diagnostic plots revealed that none of the studies can be considered as adequate for a parametric analysis, as shown in Supplementary Fig. 3a–f.

Moreover, analysis of PFS1 and PFS2 dependence showed that Kendall’s *τ* ranged from 0.17 to 0.35, highlighting an overall positive though weak correlation (see Supplementary Fig. 4a–f).

PFSratio analysis was then performed with all five methods described above. First, we explored whether *S*_PFSratio_(*δ*) estimation was consistent between the methods with different *δ* values (ranging from 0.1 to 3.0). Supplementary Fig. 5a–f plotted the *S*_PFSratio_(*δ*) curve based on different methods and Supplementary Table 1 reported the values of *S*_PFSratio_(*δ*) at *δ*=1.3, 1.5 and 2.0.

**Fig. 3:**
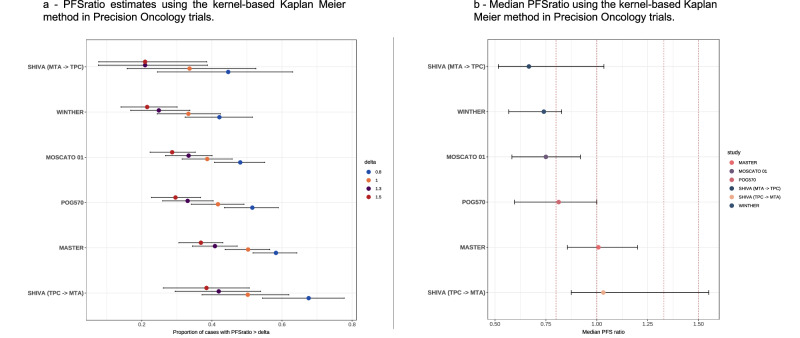
PFSratio-based comparison of precision oncology clinical trials. **a** S_PFSratio_(δ), **b** median PFSratio Vertical dotted lines identify 0.8, 1, 1.3, and 1.5 points on the x axis. Abbreviations: MTA molecularly targeted agent, PFS progression free survival, PFSratio progression free survival ratio, TPC treatment of physician’s choice.

The kernel-based Kaplan Meier method, being asymptotically unbiased, served as a reference for comparison. The curve based on the parametric method deviated considerably from that based on the kernel-based Kaplan Meier method in most studies, likely due to the violation of the parametric assumption. For the count-based, Kaplan Meier and midrank methods, which are all non-parametric, curves were very close to that based on the kernel-based Kaplan Meier method in studies with censoring rate < 10%, including WINTHER, MASTER, MOSCATO 01, and SHIVA (MTA - > TPC). However, differences became non-negligible in studies with censoring rate > 10%, including POG570 (20% censoring) and SHIVA (TPC - > MTA) (13% censoring). The count-based method substantially underestimated *S*_PFSratio_(*δ*) for *δ* > 1.3 in the SHIVA (TPC - > MTA) study. The midrank method overestimated *S*_PFSratio_(*δ*) in the SHIVA (TPC - > MTA) study, and substantially overestimated *S*_PFSratio_(*δ*) in the POG570 study. The Kaplan Meier method overestimated *S*_PFSratio_(*δ*) for *δ* > 1 in the POG570 study. A visual comparison of trials’ *S*_PFSratio_(*δ*) and median PFSratio, as evaluated with the kernel-based Kaplan Meier method, is shown in Fig. 3a, b and Supplementary Fig. 6a–f.

The plots highlight that MASTER and the TPC - > MTA arm of SHIVA were associated with the best *S*_PFSratio_(*δ*) and median PFSratio.

We also explored the impact of adopting the modified PFSratio, for *δ*=1.3 and a minimum satisfying PFS2 of 6 months. Supplementary Table 1 shows that this method provides a slightly higher *S*_PFSratio_(1.3) for WINTHER, MASTER and POG570 and a very similar or even smaller estimate for MOSCATO 01 and SHIVA, which is due to the high proportion of cases with PFS1 < 2 months in these trials.

Finally, given the weak correlation between PFS1 and PFS2 that we observed in the five studies, we performed a GBVE-based exploratory analysis aimed at evaluating the effective power of the PFSratio-based analysis and the sample size that would have been required to obtain a power of 90%. To this aim, we set *δ*_1_ at 1.3 and 1.5, while *δ*_0_ at 0.8 and 1, the expected proportion of non-informative pairs at 10% and we assumed Kendall’s *τ* as the *ρ* index of power/sample size calculation formula. As shown in Supplementary Table 2, most trials were adequately sized for a PFSratio-based analysis with *δ*_1_ at 1.3 and *δ*_0_ at 0.8, which represents the most rational clinical scenario. However, if *δ*_0_ is set at 1 (or higher), with the observed PFS1-PFS2 correlations, the PFSratio-based analysis would be clearly underpowered, and much larger sample sizes would be required.

## Discussion

The main advantage of using PFSratio as a primary endpoint in clinical trials is the possibility of designing studies with good homogeneity (as each patient acts as a control of him/herself) and with a limited sample size. This is indeed useful in PO studies, such as basket, umbrella or N-of-1 studies^2,22^ in which each entity (patient, tumor type, target molecular alteration and targeted treatment) is rare and would therefore pose an accrual and time challenge in case of large, randomized studies. In the last 10 years, several molecularly driven clinical trials have demonstrated the potential to increase the therapeutic possibilities for patients with advanced cancers, with many adopting PFSratio as primary or secondary endpoint^23^. However, most trials did not adopt proper methods for study design and analysis of paired survival times, in part because statistical properties of this endpoint have not been widely explored. For example, among studies considered here, both MOSCATO 01 and WINTHER had PFSratio as primary endpoint, but by testing the *S*_PFSratio_(*δ*) against an arbitrary proportion, the authors concluded insufficient activity.

In this work, we summarized methods for PFSratio-based clinical trials design and analysis, so that these could be adopted in future studies. With regards to the analytical phase, among the five methods described, we consider the kernel-based Kaplan Meier as the most recommended, especially in contexts where the percentage of censored cases is high ( > 20%), as it is intuitive for clinicians, able to provide an estimate of the median PFSratio and to account for dependent censoring. In the kernel-based Kaplan-Meier estimator, the use of a two-stage procedure combined with a kernel method strengthens its robustness and validity, as i) it explicitly handles the inherent dependent censoring of the PFSratio by conditioning on PFS1, enabling unbiased, fully nonparametric estimation of *S*_PFSratio_(*δ*), ii) with a modified Silverman kernel, it ensures numerical stability under right-skewed PFS1 and iii) it results in systematically better calibrated curves than KM/naïve parametric estimates, with differences amplified with increasing censoring. Contrarily, despite its immediacy, the count-based method does not consider PFSratio as a time-to-event variable and thus ignores the impact of censored pairs. As such, it requires exclusion of cases with PFSratio ≤ *δ* if censored, while cases with PFSratio > *δ* are kept as successful irrespective of the censoring status. Therefore, we consider this method as the least attractive, and it should not be adopted in settings with high censoring rates, as it may lead to incorrect exclusion of a significant number of patients from the analysis. The parametric method is limited by the assumption that PFS1 and PFS2 data need to follow a Weibull distribution, which is not always fulfilled. Nonetheless, we showed how overall results are very similar with the different methods in the five studies explored.

Concerning trial design, we implemented a user-friendly tool to calculate the required sample size (or the study power, if the sample is given) directly providing an expected GTE or evaluating it with two different methods (GBVE and Weibull). Among these, the GBVE method is influenced by the expected correlation between PFS1 and PFS2, but grants the design of PFSratio-based trial with adequate power and limited sample size in most cases. In our opinion, in a PO setting, a *δ*_1_ > 1 (1.3 or 1.5, i.e., the molecularly informed treatment is expected to perform better than patient’s last prior therapy) and *δ*_0_ < 1 (i.e. if not effective, the molecularly informed treatment is expected to perform worse than patient’s last prior therapy, as PFS is expected to deteriorate with subsequent lines of therapy) represents the optimal design to conduct a clinical trial on a N-of-1 population.

Given these tools, we performed a PFSratio-based survey of five PO clinical clinical trials. First, our analysis highlighted a relevantly weak PFS1/PFS2 correlation in all studies, which might be due to the heterogeneity of tumor types and corresponding treatments included in each study. In this light, future studies are needed to elucidate which cancer types are associated with strongest intra-patient correlation and can thus be studied with small sample-sized, PFSratio-based clinical trials. Until such data become available, PO trials should be designed assuming a weak to moderate *ρ*.

Furthermore, we compared PFSratio-based results between trials as assessed by the kernel-based Kaplan Meier method. This analysis revealed POG570, MASTER and the TPC - > MTA SHIVA arm as studies with higher *S*_PFSratio_(*δ*) and median PFSratio. While a proper comparison between these trials cannot be done, we can hypothesize some reasons for this difference: first, while these trials were overall conducted in a similar time frame, MOSCATO 01 and WINTHER results were published much earlier, so that targeted therapies options in these trials may have been limited compared to more recent studies. More likely, POG570 and MASTER patients were treated according to results of WGS/WES and transcriptome sequencing, as compared to targeted sequencing panels of the other studies, which may have provided more informative results for targeted therapies. Also, MASTER selectively includes patients with rare cancers or cancers in young patients, where molecularly informed therapies can have more impact as standard therapeutic options are even more limited. Finally, a separate consideration must be done for SHIVA, given the study design (see below) and since MTAs associated with longer PFSratio were represented by hormone therapies (tamoxifen or abiraterone) or everolimus, thus only partially comparing to other studies^18^.

PFSratio has limitations as primary endpoint for clinical trials. First, it must be noted that PFS2 is calculated from the first day of treatment administration, thus not considering the time that may elapse from the end of the prior treatment and the impact this may have on patients’ status. Moreover, PFSratio has been mostly adopted in PO trials performed in patients progressing after exhaustion of standard treatment options, which may have provided the greatest OS benefit. Based on this, the PFSratio cannot be adopted as a granted surrogate endpoint for OS, and its exploration in this regard has been encouraging but limited^17^ so far.

Most importantly, a reliable estimation of PFSratio is based on uniform disease assessment during the two lines of treatment considered, both in terms of frequency (e.g., CT scans regularly performed every 2 months)^8^ and of techniques (radiological, clinical or laboratory assessment methods), as suggested in Fig. 1. This is questionable for both PFS1 and PFS2 in retrospective studies: in a recent meta-analysis on outcomes of treatments recommended by molecular tumor boards, Gladstone et al. highlighted the high variation in the required period to fulfill the criteria for defining stable disease, ranging from 6 weeks to 6 months, among the studies considered^23^, potentially resulting in poor interpretability of PFSratio. Similarly, clinical trials adopting PFSratio have frequently considered PFS1 retrospectively, which can result in estimation bias. For example, CT scans during prior standard treatment may have been performed at wider intervals, resulting in artificial lengthening of PFS1. Conversely, PFS1 may be assessed in the context of clinical trials with shorter evaluation intervals (e.g., CT scans every 6 weeks) or defined using non-radiological criteria (e.g., increases in tumor blood biomarkers), which can lead to PFSratio being distorted in the opposite direction. In this light, the cohort of crossing-over patients from the SHIVA trial represented the optimal setting for PFSratio exploration, as both PFS1 and PFS2 were assessed within the study, so that the striking difference in median PFSratio between the two SHIVA cohorts (TPC - > MTA vs MTA - > TPC) is not surprising. Therefore, we consider it mandatory that studies reporting a PFSratio-based analysis provide detailed information on the assessment methods and timing of evaluations for both PFS1 and PFS2.

Most importantly, statistical considerations must be balanced with clinical judgment to determine the appropriateness of PFSratio use in a given context. Although random variability may arise from the weak correlation between PFS1 and PFS2, PO provides an ideal setting for the adoption of PFSratio as an endpoint. In this context, targeted therapies (captured by PFS2) are specifically expected to surpass the efficacy of preceding non-targeted treatments (PFS1). This reversal -contrary to the physiological progressive shortening of PFS with successive lines of therapy -is precisely what indicates benefit of the targeted intervention. In this light, although not an explicit requirement, the interpretability of PFSratio in PO studies implicitly relies on the similarity of non-targeted treatments used for PFS1. Also, while very short or long PFS1 may reduce the clinical relevance of PFSratio, we showed that the use of a modified PFSratio results in estimate distortions, and should thus be carefully guided by the clinical setting and according to the specific study scenario.

Finally, so far, PFSratio has been used primarily as an endpoint in single-arm studies. However, future directions include the development of suitable methodologies to compare two or more treatment arms, incorporating multivariable analyses that adjust for relevant clinical and biological patient characteristics.

In conclusion, we provided a clinician-oriented overview of PFSratio statistical properties, which can represent a benchmark for future study design and analysis. The methods presented are meant for a phase II clinical trial, especially in the PO, N-of-1 setting where each patient represents a rare entity treated with molecularly informed therapies. We applied these methods to survey five PO clinical trials, highlighting differences and pitfalls, and implemented the methods in a user-friendly web application. With these tools, prospective trials are awaited to confirm the validity of PFSratio as a clinically meaningful endpoint in PO.

## Methods

The manuscript is meant as a guide for a reader with a clinical background in medical oncology and therefore avoid an in-depth statistical description with mathematical formulae, for which reference is made to dedicated works^4–7,15,16^. Stemming from these works, we wrapped and implemented the methods here presented in the ***PROPHETS*** R package and Shiny app (https://github.com/fedenichetti/prophets_package) as a user-friendly web calculator for clinical purposes. All analyses later shown were performed in R (Version 4.2.0, 2022-04-22) using the RStudio software [Posit open source data science company].

## Supplementary information

Supplementary Information

## Data Availability

Paired survival outcomes of the WINTHER^12^ and MOSCATO 01^11^ study are publicly available from original publications. For SHIVA^21^, MASTER^17^ and POG570^19^, data are available upon reasonable request shared to the corresponding author and to corresponding authors of the original publications.

## Code availability

The code for the ***PROPHETS*** R package and Shiny app are available at https://github.com/fedenichetti/prophets_package.
