# Supplementary material for: Benchmarking progression-free survival ratio as primary endpoint in precision oncology clinical trials
Source: NPJ Precis Oncol. 2025 Dec 15;10:38. doi: 10.1038/s41698-025-01231-x (PMC12820104; doi:10.1038/s41698-025-01231-x)

Supplementary Data

Supplementary Tables

Supplementary Table 1. Summary of PFSratio and modified PFSratio (mPFSratio) analysis results in precision oncology clinical trials, using five different methods (count-based, Kaplan Meier, kernel-based Kaplan Meier, parametric, and Midrank). $S_{PFSratio}(\delta)$ values are reported for

each trial and method at δ thresholds of 1.3, 1.5, and 2.0, along with corresponding 95% confidence intervals (shown in brackets). Results are shown for both the original and modified versions of the PFS ratio proposed in the manuscript.

Method		δ	WINTHER	MASTER	MOSCATO 01	POG570	SHIVA (TPC -> MTA)	SHIVA (MTA -> TPC)
**Count-based**	PFSratio	1.3	0.25 (0.17 - 0.34)	0.40 (0.34 - 0.47)	0.34 (0.27 - 0.41)	0.31 (0.24 - 0.39)	0.39 (0.27 - 0.53)	0.21 (0.07 - 0.42)
	mPFSratio		0.27 (0.19 - 0.37)	0.43 (0.37 - 0.50)	0.32 (0.26 - 0.39)	0.39 (0.32 - 0.47)	0.33 (0.21 - 0.46)	0.21 (0.07 - 0.42)
**Kaplan Meier**	PFSratio		0.25 (0.18 - 0.35)	0.41 (0.35 - 0.48)	0.34 (0.28 - 0.41)	0.35 (0.28 - 0.43)	0.44 (0.33 - 0.58)	0.21 (0.10 - 0.45)
	mPFSratio		0.27 (0.20 - 0.37)	0.45 (0.39 - 0.51)	0.33 (0.27 - 0.40)	0.41 (0.34 - 0.49)	0.36 (0.26 - 0.50)	0.21 (0.10 - 0.45)
**KernelKM**	PFSratio*		0.25 (0.17 - 0.34)	0.41 (0.34 - 0.47)	0.33 (0.27 - 0.40)	0.33 (0.26 - 0.40)	0.42 (0.29 - 0.54)	0.21 (0.07 - 0.39)
	mPFSratio		0.27 (0.19 - 0.36)	0.45 (0.38 - 0.51)	0.33 (0.26 - 0.40)	0.42 (0.35 - 0.49)	0.34 (0.22 - 0.46)	0.21 (0.07 - 0.39)
**Midrank**	PFSratio		0.24 (0.16 - 0.32)	0.41 (0.35 - 0.47)	0.34 (0.27 - 0.40)	0.38 (0.32 - 0.45)	0.44 (0.33 - 0.56)	0.20 (0.04 - 0.36)
	mPFSratio		0.27 (0.19 - 0.36)	0.44 (0.38 - 0.50)	0.33 (0.26 - 0.40)	0.40 (0.33 - 0.47)	0.40 (0.29 - 0.51)	0.20 (0.04 - 0.36)
**Parametric**	PFSratio		0.26 (0.16 - 0.36)	0.44 (0.37 - 0.51)	0.32 (0.24 - 0.40)	0.38 (0.30 - 0.47)	0.47 (0.34 - 0.60)	0.25 (0.01 - 0.48)
	mPFSratio		0.24 (0.14 - 0.34)	0.42 (0.35 - 0.49)	0.27 (0.20 - 0.35)	0.38 (0.29 - 0.46)	0.40 (0.26 - 0.53)	0.22 (-0.01 - 0.46)
**Count-based**	PFSratio	1.5	0.21 (0.13 - 0.30)	0.36 (0.30 - 0.43)	0.30 (0.23 - 0.37)	0.28 (0.22 - 0.36)	0.33 (0.21 - 0.46)	0.21 (0.07 - 0.42)
	mPFSratio		0.27 (0.19 - 0.37)	0.40 (0.34 - 0.47)	0.26 (0.20 - 0.33)	0.36 (0.29 - 0.44)	0.30 (0.19 - 0.43)	0.21 (0.07 - 0.42)
**Kaplan Meier**	PFSratio		0.21 (0.15 - 0.30)	0.37 (0.32 - 0.44)	0.29 (0.23 - 0.36)	0.32 (0.25 - 0.40)	0.40 (0.29 - 0.54)	0.21 (0.10 - 0.45)
	mPFSratio		0.27 (0.20 - 0.37)	0.42 (0.36 - 0.49)	0.27 (0.22 - 0.34)	0.38 (0.32 - 0.47)	0.34 (0.24 - 0.48)	0.21 (0.10 - 0.45)
**KernelKM**	PFSratio		0.22 (0.14 - 0.30)	0.37 (0.30 - 0.43)	0.29 (0.22 - 0.35)	0.30 (0.23 - 0.37)	0.38 (0.26 - 0.51)	0.21 (0.08 - 0.39)
	mPFSratio		0.27 (0.19 - 0.36)	0.42 (0.36 - 0.49)	0.28 (0.21 - 0.34)	0.39 (0.32 - 0.47)	0.32 (0.20 - 0.44)	0.21 (0.07 - 0.39)
**Midrank**	PFSratio		0.21 (0.13 - 0.28)	0.37 (0.31 - 0.43)	0.29 (0.23 - 0.36)	0.34 (0.27 - 0.41)	0.41 (0.30 - 0.53)	0.20 (0.04 - 0.36)
	mPFSratio		0.27 (0.19 - 0.36)	0.42 (0.36 - 0.48)	0.28 (0.22 - 0.35)	0.38 (0.31 - 0.45)	0.39 (0.27 - 0.50)	0.20 (0.04 - 0.36)
**Parametric**	PFSratio		0.22 (0.11 - 0.33)	0.39 (0.31 - 0.47)	0.28 (0.19 - 0.37)	0.35 (0.25 - 0.44)	0.41 (0.26 - 0.56)	0.20 (-0.06 - 0.46)
	mPFSratio		0.21 (0.10 - 0.31)	0.37 (0.29 - 0.45)	0.24 (0.15 - 0.32)	0.34 (0.24 - 0.44)	0.34 (0.18 - 0.49)	0.17 (-0.08 - 0.43)
**Count-based**	PFSratio	2.0	0.12 (0.07 - 0.20)	0.30 (0.24 - 0.36)	0.21 (0.16 - 0.28)	0.23 (0.17 - 0.30)	0.23 (0.13 - 0.36)	0.12 (0.03 - 0.32)
	mPFSratio		0.23 (0.16 - 0.33)	0.34 (0.28 - 0.41)	0.20 (0.15 - 0.27)	0.33 (0.26 - 0.40)	0.22 (0.12 - 0.35)	0.17 (0.05 - 0.37)
**Kaplan Meier**	PFSratio		0.12 (0.07 - 0.21)	0.31 (0.26 - 0.38)	0.23 (0.17 - 0.29)	0.25 (0.19 - 0.33)	0.31 (0.20 - 0.46)	0.12 (0.04 - 0.36)
	mPFSratio		0.23 (0.17 - 0.33)	0.36 (0.30 - 0.43)	0.22 (0.17 - 0.29)	0.35 (0.28 - 0.43)	0.26 (0.17 - 0.40)	0.17 (0.07 - 0.41)
**KernelKM**	PFSratio		0.13 (0.07 - 0.20)	0.31 (0.25 - 0.37)	0.22 (0.16 - 0.28)	0.23 (0.17 - 0.30)	0.30 (0.18 - 0.43)	0.13 (0.03 - 0.30)
	mPFSratio		0.23 (0.16 - 0.32)	0.36 (0.30 - 0.42)	0.22 (0.16 - 0.28)	0.36 (0.28 - 0.44)	0.24 (0.14 - 0.36)	0.16 (0.05 - 0.34)
**Midrank**	PFSratio		0.12 (0.06 - 0.18)	0.31 (0.26 - 0.37)	0.23 (0.17 - 0.29)	0.29 (0.22 - 0.35)	0.33 (0.22 - 0.44)	0.12 (-0.01 - 0.25)
	mPFSratio		0.23 (0.15 - 0.31)	0.36 (0.30 - 0.42)	0.23 (0.17 - 0.29)	0.34 (0.27 - 0.41)	0.30 (0.19 - 0.41)	0.16 (0.02 - 0.30)
**Parametric**	PFSratio		0.16 (0.03 - 0.28)	0.29 (0.19 - 0.40)	0.21 (0.09 - 0.32)	0.28 (0.16 - 0.41)	0.29 (0.10 - 0.48)	0.12 (-0.18 - 0.42)
	mPFSratio		0.16 (0.03 - 0.28)	0.28 (0.18 - 0.39)	0.17 (0.07 - 0.28)	0.28 (0.15 - 0.41)	0.23 (0.03 - 0.44)	0.11 (-0.17 - 0.39)

*corresponds to the $S_{PFSratio}$ reported in the last column of Table 2.

Abbreviations: KernelKM: Kernel-based Kaplan Meier; MTA: molecularly targeted agent; (m)PFSratio: (modified) progression free survival ratio; TPC: treatment of physician's choice; $S_{PFSratio}$: the probability that PFSratio is equal to or greater than δ.

Supplementary Table 2. PFSratio-based analysis of precision oncology trials design.

	Kendall's Tau	$H_1(\delta)$	$H_0(\delta)$	Sample size	Corresponding Power	Required Sample Size with 90% Power
WINTHER	0.314	1.5	1.0	107	82	152
		1.3	1.0		47	353
		1.5	0.8		99	68
		1.3	0.8		92	109
MASTER	0.220	1.5	1.0	255	98	187
		1.3	1.0		74	436
		1.5	0.8		99	82
		1.3	0.8		99	133
MOSCATO 01	0.174	1.5	1.0	194	91	206
		1.3	1.0		58	481
		1.5	0.8		99	67
		1.3	0.8		98	109
POG570	0.228	1.5	1.0	190	93	184
		1.3	1.0		62	428
		1.5	0.8		99	81
		1.3	0.8		98	131
SHIVA (TPC -> MTA)	0.295	1.5	1.0	70	62	159
		1.3	1.0		32	369
		1.5	0.8		92	71
		1.3	0.8		77	113

Based on resulting PFS1-2 correlation (Kendall's Tau) and pre-defined alternative and null PFSratio cutoffs, the study powers are calculated according to the GBVE model and the real sample sizes are compared to the required ones if PFSratio was adopted as primary endpoint with 90% power.

Abbreviations: $H_{0/1}(\delta)$: null/alternative hypothesis delta; MTA: molecularly targeted agent; TPC: treatment of physician's choice.

Supplementary Figures

Supplementary Figure 1(a-b). Graphic representation of the impact of correlation (rho), sample size, HR on power in PFSratio-based clinical trial design.

Abbreviations: GBVE=Gumbel's type B bivariate extreme-value; kappa= shape of the hazard function; rho= pearson correlation index between PFS1 and PFS2; R = median ratio between PFS1 and 2, detailed with corresponding δ_1 and δ_0.

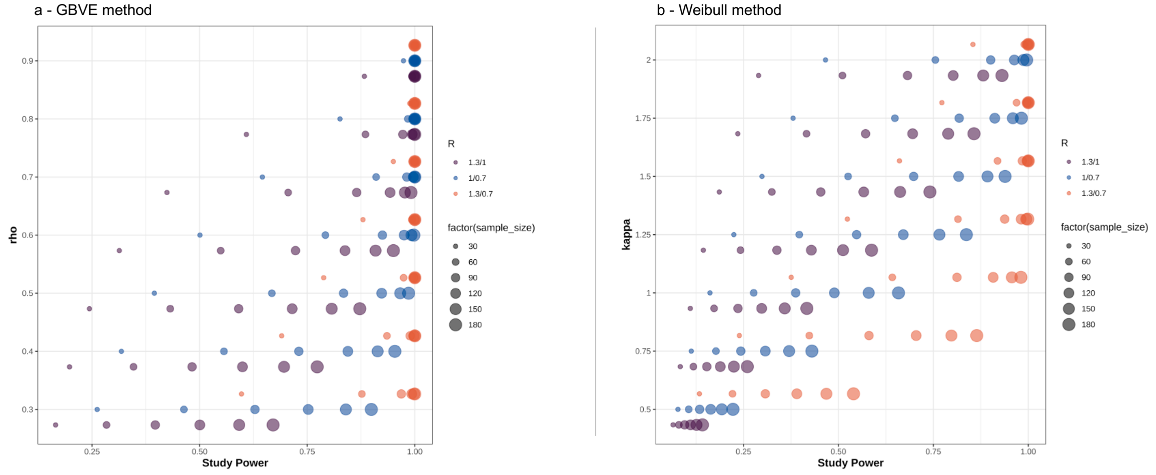

Supplementary Figure 2(a-f). Graphical representation of individual patient PFS1 and PFS2 in precision oncology clinical trials. Dots at top of each bar denote censoring status; red and grey dots represent censored (=0, no disease progression on PFS2) and not censored (=1, disease progression was documented on PFS2), respectively. Patients above the red horizontal dotted line have PFSratio equal to or greater than δ, here set at 1.3.

Abbreviations: MTA: molecularly targeted agent; PFS1/PFS2: progression free survival 1/2; PFSratio: progression free survival ratio; TPC: treatment of physician's choice.

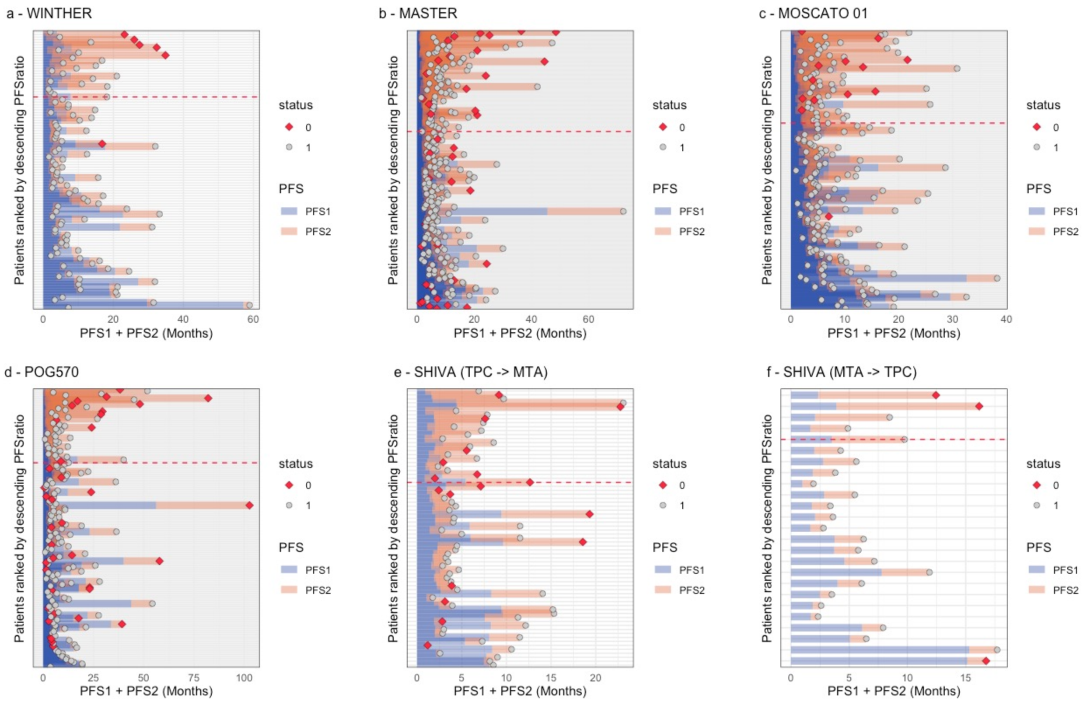

Supplementary Figure 3(a-f). Weibull regression diagnostic plots of precision oncology clinical trials. The Weibull model would be appropriate if the plots would show straight, parallel lines. Crosses represent censoring status of patients.

Abbreviations: MTA: molecularly targeted agent; PFS1/PFS2: progression free survival 1/2; TPC: treatment of physician's choice.

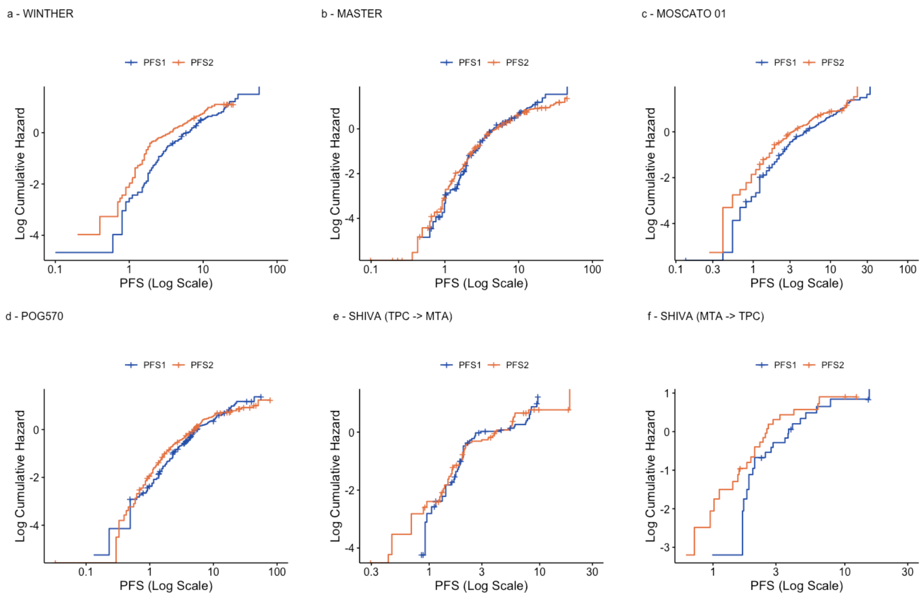

Supplementary Figure 4(a-f). Scatter plot of individual PFS1 (x axis) and PFS2 (y axis) of precision oncology clinical trials. The size of the dots corresponds to PFSratio, with largest dots appearing in the upper-left quadrant of the plot, corresponding to cases with longer PFS2 and shorter PFS1. Orange dots denote patients with PFSratio > 1.3.

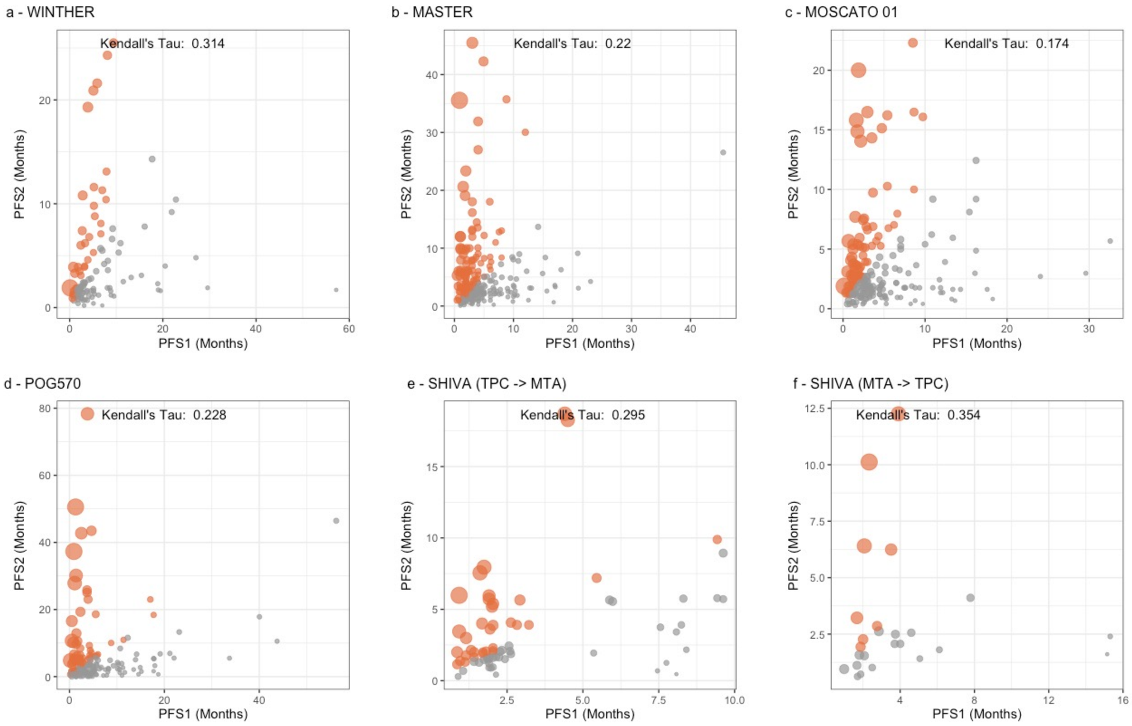

Supplementary Figure 5(a-f). Results of the PFSratio-based analysis across a range of δ values (from 0.1 to 3) using five different methods (count-based, Kaplan Meier, kernel-based Kaplan Meier, parametric, and Midrank) applied to precision oncology clinical trials. Each point represents the $S_{PFSratio}(\delta)$ (y-axis) for a specific δ (x-axis), calculated using one of the five methods. Points corresponding to the same method are color-coded and connected to illustrate how estimates vary across δ values.

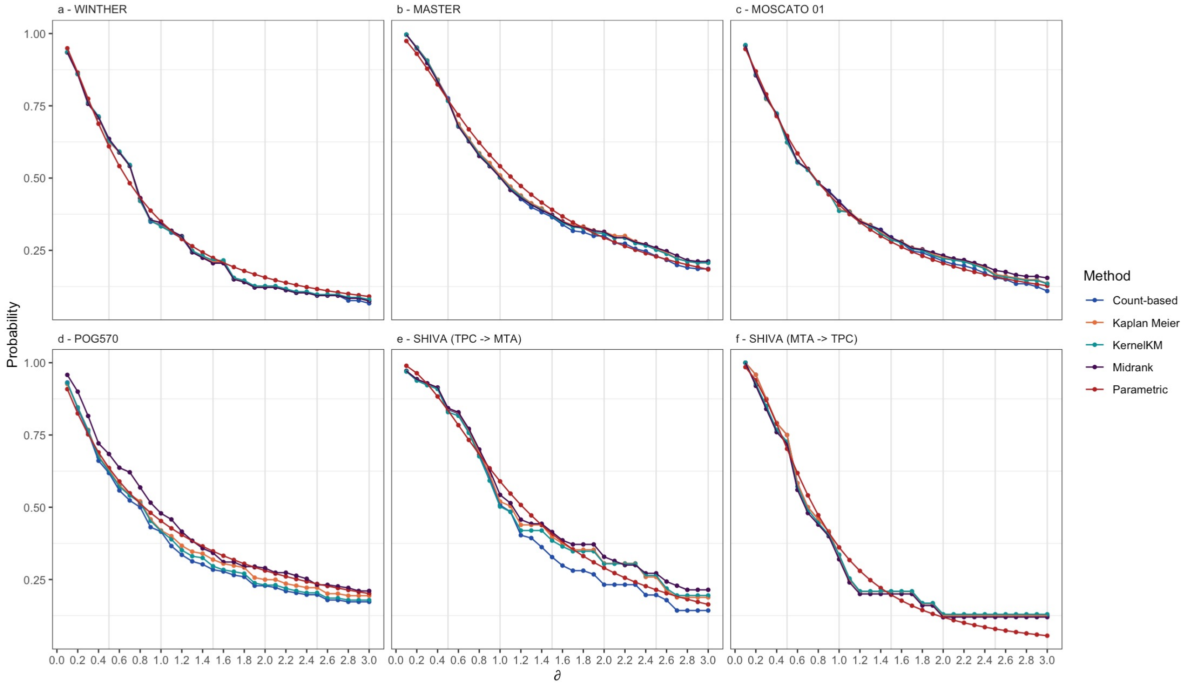

Supplementary Figure 6(a-f). Kernel-based Kaplan Meier curves of PFSratio of precision oncology clinical trials. The x axis represents levels of δ, the y axis represents the S_{PFSratio}, i.e. the probability that PFSratio is equal to or greater than δ.

Abbreviations: MTA: molecularly targeted agent; PFSratio: progression free survival ratio; TPC: treatment of physician's choice.

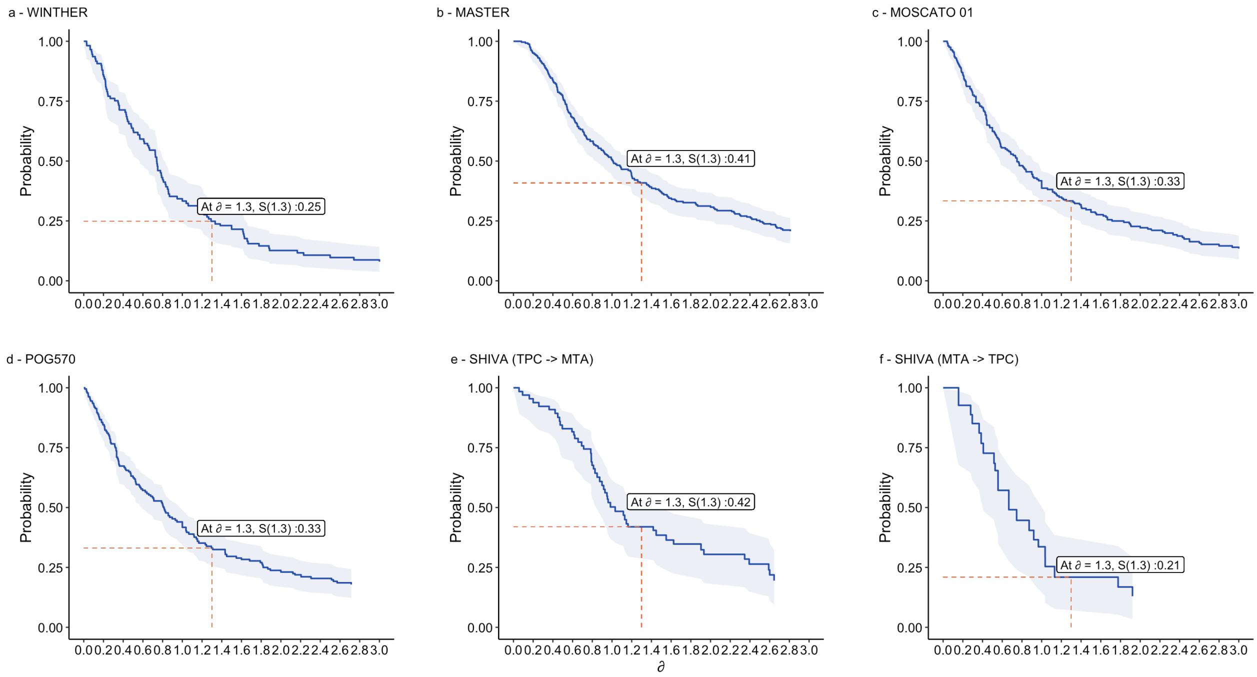

Supplement: Supplementary file 1 — Supplementary Information [file 41698_2025_1231_MOESM1_ESM.pdf]
